# Supplementary material for: Digital transformation and total factor productivity: Empirical evidence from China
Source: PLoS One. 2023 Oct 16;18(10):e0292972. doi: 10.1371/journal.pone.0292972 (PMC10578585; doi:10.1371/journal.pone.0292972)
Supplement: Attachment — Submitted filename: Response to Reviewers.docx [file pone.0292972.s002.docx]

**Response to Reviewers**

We would like to express our heartfelt gratitude to three anonymous reviewers for their valuable feedback. Our manuscript, titled “**Digital Transformation and Total Factor Productivity: Empirical Evidence from China**”, benefited significantly from the constructive comments and insights from the review team. Based on the suggestions received, we have made careful revisions to the original manuscript. In the revised manuscript, all changes are marked in red. In addition, we have also carefully proofread this manuscript for typographical, grammatical, and other errors. We hope the revised manuscript is able to meet your standard of quality and address the concerns raised by the reviewers. In the following, you will find our detailed point-by-point responses to the reviewers’ comments.

**Reviewer #2**

**Major Comments**

**The authors did an excellent work revising the paper and I very much appreciate their efforts. Now the construction of IVs makes more sense than the original version. In my initial comments, I was concerned that the baseline results (OLS results) are significantly different from those obtained from IV regressions, indicating the OLS approach may suffer from omitted variable bias (endogeneity issues). The authors adopted IVs for heterogeneity and mechanism analysis, which is fine. But I still recommend the authors briefly discuss the differences between OLS estimates and IVs estimates and make it clear that the endogeneity issue is indeed a threat to their estimation and thus they use IVs in later sections.**

***Response：***

Thank you for your valuable comments, which help us a lot. The differences between OLS and IVs estimation are briefly discussed in the paper, in addition to the fact that we have made it clear in the paper that the endogeneity problem does threaten our estimation results, so we use IVs in the later sections. The specific alteration is as follows:

…

Line 304

It should be emphasized that the IVs estimates obtained in this paper through instrumental variables are local average treatment effects that exclude the part of exogenous changes included in the explanatory variable digital transformation. That is, changes in firms' digital transformation due to bidirectional causation are excluded, and thus the regression coefficients are significantly different from the OLS coefficient values. In addition, because the endogeneity issue does affect the study estimates, we use IVs estimation in both the heterogeneity test and the mechanism test later.

…
